# The Potential Use of Peptides in the Fight against Chagas Disease and Leishmaniasis

**DOI:** 10.3390/pharmaceutics16020227

**Published:** 2024-02-04

**Authors:** Hayelom Berhe, Mahesh Kumar Cinthakunta Sridhar, Mulate Zerihun, Nir Qvit

**Affiliations:** The Azrieli Faculty of Medicine in the Galilee, Bar-Ilan University, Safed 1311502, Israel; hayelom.berhe8@gmail.com (H.B.); mahesh1996s@gmail.com (M.K.C.S.); mulatezerihun@yahoo.com (M.Z.)

**Keywords:** neglected tropical diseases, parasites, Chagas disease, leishmaniasis, life cycle, drug target, biochemical mechanisms, pharmacological therapeutics, peptides

## Abstract

Chagas disease and leishmaniasis are both neglected tropical diseases that affect millions of people around the world. Leishmaniasis is currently the second most widespread vector-borne parasitic disease after malaria. The World Health Organization records approximately 0.7–1 million newly diagnosed leishmaniasis cases each year, resulting in approximately 20,000–30,000 deaths. Also, 25 million people worldwide are at risk of Chagas disease and an estimated 6 million people are infected with *Trypanosoma cruzi*. Pentavalent antimonials, amphotericin B, miltefosine, paromomycin, and pentamidine are currently used to treat leishmaniasis. Also, nifurtimox and benznidazole are two drugs currently used to treat Chagas disease. These drugs are associated with toxicity problems such as nephrotoxicity and cardiotoxicity, in addition to resistance problems. As a result, the discovery of novel therapeutic agents has emerged as a top priority and a promising alternative. Overall, there is a need for new and effective treatments for Chagas disease and leishmaniasis, as the current drugs have significant limitations. Peptide-based drugs are attractive due to their high selectiveness, effectiveness, low toxicity, and ease of production. This paper reviews the potential use of peptides in the treatment of Chagas disease and leishmaniasis. Several studies have demonstrated that peptides are effective against Chagas disease and leishmaniasis, suggesting their use in drug therapy for these diseases. Overall, peptides have the potential to be effective therapeutic agents against Chagas disease and leishmaniasis, but more research is needed to fully investigate their potential.

## 1. Introduction

Chagas disease (CD), leishmaniasis, and human African trypanosomiasis (HAT) are neglected tropical diseases (NTDs) caused by insect vector-borne protozoan parasites. Over 100,000 people die each year from NTDs worldwide, most living on less than USD 2 per day [1]. Chagas disease (aka American trypanosomiasis) is an infectious disease caused by the parasite *Trypanosoma cruzi* (*T. cruzi*), African Trypanosomiasis (“sleeping sickness”) is caused by *Trypanosoma brucei* (*T. brucei*), and leishmaniasis is caused by a protozoa parasite from over 20 *Leishmania* species [2,3]. Leishmaniasis is ranked second among all protozoan diseases for mortality after malaria [4]. The current drugs used against NTDs are suboptimal. For example, the only Food and Drug Administration (FDA)-approved medications for leishmaniasis are intravenous liposomal amphotericin B (L-AmB) for visceral leishmaniasis (VL) and oral miltefosine for cutaneous leishmaniasis (CL), mucosal leishmaniasis (ML), and VL caused by particular species [5]. The cost of treatment with amphotericin B deoxycholate varies widely between countries, ranging from less than USD 1 to USD 171 per day. Liposomal amphotericin B is a newer formulation that is less toxic but more expensive than deoxycholate amphotericin B. The cost of a single dose of 50 mg amphotericin B deoxycholate varies widely across countries. In Zambia, the Netherlands, Russia, and Chile, it is less than USD 1 per day. In India, prices range from USD 1 to USD 10 per dose, while in the United States (US), a single dose costs approximately USD 50 [6,7]. Amphotericin B deoxycholate is well known for its severe and potentially lethal side effects (e.g., fever, shaking, chills, flushing, loss of appetite, and many more). As a result, the recommended course of treatment is frequently not followed, resulting in the development of resistance [8,9,10]. Moreover, there are no effective vaccines for leishmaniasis [10]. Similar challenges are also associated with drugs for Chagas disease. The safety profile of the currently available drugs is far from ideal, with frequent adverse events and high drug discontinuation rates. There are only two drugs available for treating Chagas disease: benznidazole (BZN) and nifurtimox (NFX). Although these drugs are effective in the acute phase, they have limitations for chronic infections, and both drugs are restricted due to their toxic effects. For example, Nifurtimox is no longer used in many places since it causes neurological disorders or psychiatric episodes. Therefore, there is an urgent need to develop effective, safe, and affordable drugs [11]. 

Drug discovery aims to identify novel compounds that specifically and significantly change the course of the diseases they are intended to treat. There is a pressing need to identify new therapeutics for NTDs and novel approaches to drug design and development are needed. However, due to the lack of commercial benefits, pharmaceutical companies are discouraged from investing in NTD product development [12,13]. Recently, virtual screening, a convenient and cost-effective solution in the early stages of drug discovery, has been used for the discovery of anti-trypanosomal and anti-leishmanial drugs with some promising results [14]. Nevertheless, computational methods should be validated experimentally [15]. Other examples are target-based or phenotype-based drug discovery, which are probably the most effective strategies for drug discovery for NTDs [16]. Another appealing approach is repurposing existing drugs. Drug repurposing discovers new disease indications for previously approved drugs that benefit from accessible compound libraries. This has yielded some promising compounds for various NTDs. Yet, repurposing an existing drug poses a major challenge in identifying an appropriate therapeutic indication for it. It involves multiple factors, such as technology, patents, investment, and clinical trials. Recent advances in production, modification, and analytical technologies have led to an increased interest in peptides as therapeutics for NTDs [17,18]. 

## 2. Leishmaniasis and Chagas Disease

### 2.1. Life Cycle of Trypanosomatidae 

**Figure 1:**
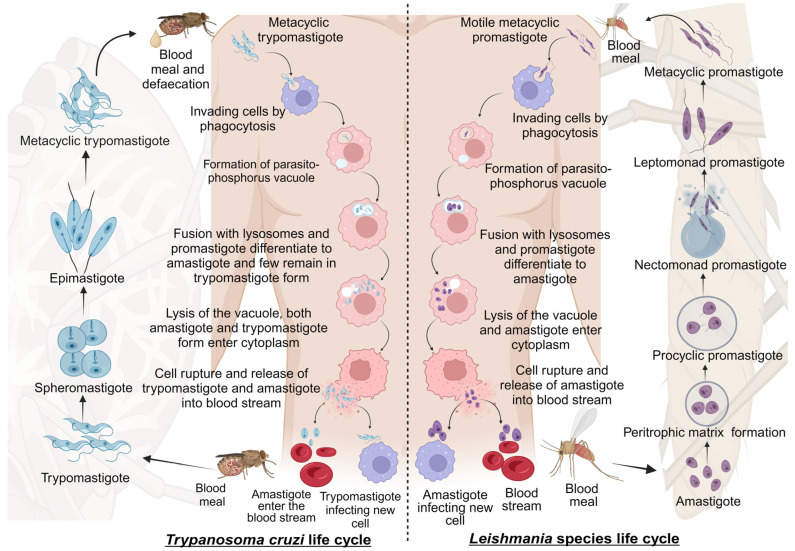
The life cycle of *Leishmania* species and *Trypanosoma cruzi*. The figure elaborates the similarities and differences between the two parasitic infestations involving the vector, host, and stages of parasitic differentiation. Though there are few similarities in the parasitic differentiation of Chagas and leishmaniasis in the host cells, the figure emphasizes parasitic differentiation in the vector. During a blood meal, it is also transmitted to the host. When insects feed on blood, they release feces that contain metacyclic trypomastigotes. A bite wound or conjunctiva allows the parasite to enter the bloodstream. When infiltrating the bloodstream, *T. cruzi* can infect any nucleated cell. Once the parasite enters a host cell and transforms into an amastigote, it reproduces in the cytoplasm. An intracellular parasite matures into a trypomastigote after several replications. When a mammalian host is disrupted, trypomastigotes are released into the bloodstream and ingested by triatomines during a blood meal. Epimastigotes and metacyclic trypomastigotes are present in the insect gut at various developmental stages. The figure also distinguishes between various steps involved in parasite development inside the cellular compartments and provides a complete life cycle illustration with a comparison of the life cycle of the parasites inside the vector and the host. BioRender.com (accessed on 25 September 2023) was used to generate this Figure.

Leishmaniasis and Chagas disease are caused by trypanosomatid parasites. In mammals, leishmaniasis and Chagas disease are transmitted through the sandfly and the triatomine bug, respectively. The differentiation of these trypanosomatids occurs partially in the vector and the host. This is accomplished with different developmental stages giving rise to the infective stage of the parasite that infects mammals [19]. We discuss the stages of development and differentiation of the parasites in the vector and host involved in the life cycles of both leishmaniasis and Chagas disease (Figure 1).

#### 2.1.1. Life Cycle of *Leishmania* Species 

Leishmaniasis is caused by the protozoan parasite of the genus *Leishmania* belonging to the Trypanosomatidae family. It is spread through female sandflies of the genera *Phlebotomus* and *Lutzomyia* [20]. The parasite’s life cycle is complex as it includes vector and mammalian reservoirs for its morphogenesis; hence, it resembles a digenetic life cycle [21]. The parasite’s entry is during the blood meal event, where motile metacyclic promastigotes are transferred into the blood through sandfly saliva [22]. Next, the metacyclic promastigotes migrate to healthy macrophages. Proteins on the parasite flagella interact with the macrophagic cell membrane and gain entry through phagocytosis by forming pseudopods infecting macrophages [23,24]. In the macrophage, promastigote forms differentiate from motile forms, which have elongated cell shapes and long flagellums, to amastigote forms, which have short flagellums [1]. After fusing with the parasitophorous vacuole, the host lysosomes create an environment for parasite replication. Finally, the macrophages rupture, and the amastigotes are released into the bloodstream [22]. 

The blood meal is digested in the sandfly midgut where the amastigotes cluster together forming nest cells. The nest cells are surrounded by an enclosed structure called the peritrophic matrix, which protects the amastigotes from the insect’s gut environment [22,25]. The amastigotes transform into elongated flagellated procyclic promastigotes inside the peritrophic matrix. It is their first replicative stage. After 48–72 h, the procyclic promastigotes differentiate into long motile nectomonad promastigotes that break open the peritrophic matrix and enter the midgut lumen. Nectomonad promastigotes transform into short motile nectomonad promastigotes called leptomonad promastigotes and adhere to the microvilli of the midgut epithelium of the insect [26,27]. Here, the leptomonad promastigotes migrate and concentrate at the anterior stomodeal valve. Here, they differentiate into metacyclic promastigotes that infect mammalian hosts. During the transformation to metacyclic stages, they secrete a gel called promastigote secretory gel (PSG), creating a “block fly” that forces the sandfly to feed on the blood and transfer metacyclic promastigotes from the vector to the mammalian host, thereby starting the cycle all over [28].

#### 2.1.2. Life Cycle of *Trypanosoma cruzi*

Chagas disease is a protozoan disease caused by *T. cruzi* of the genus *Trypanosoma* belonging to the Trypanosomatidae family. It is caused by the parasitic defecation of a blood-sucking vector called the triatomine bug (aka the kissing bug). As a result of the undetermined spread of feces on the wound by human actions, the parasite enters the host [29]. The first step upon entry into the host is adhesion. This is where metacyclic trypomastigotes attach to host cellular receptors with their flagella. Parasite flagella consists of molecules belonging to the Apicomplexa family that adhere to the host cell membrane. By binding glycoproteins to the cell membrane, metacyclic trypomastigotes mobilize calcium necessary for entry into the cytoplasm [30]. Next, the adhered parasite recognizes and alters ligands such as lectin-like molecules on the surface for safe internalization via endocytosis mediated by clathrin and caveolar lipids [30]. Also, internalization by phagocytosis, micropinocytosis, and circular dorsal ruffle formation has been reported [31]. The parasite resides safely within the parasite phosphorus vacuole formed by the plasma membrane and then fusses with the lysosome. When lysosomes fuse, parasites can differentiate from trypomastigotes to short globular organisms with small flagella, known as amastigotes, whereas some remain trypomastigotes [32]. After or during differentiation, the amastigotes secrete hemolysin, also called Tc-Tox, and trypomastigotes secrete trans-sialidase/neuraminidase, required for fragmentation of the parasite phosphorus vacuole, and enter the cytoplasm. As this process occurs during the differentiation of metacyclic trypomastigotes into amastigotes, both forms are observed in the cytoplasm. The intracellular amastigote form further differentiates to form bloodstream trypomastigotes. Bloodstream trypomastigotes rupture the host cell and enter the bloodstream, infecting other cells [32,33,34].

During the blood meal of an infected individual, trypomastigotes enter the triatomine bug’s gut. Here, most bloodstream trypomastigotes are digested. Surviving parasites differentiate and transform into small spherical structures called spheromastigotes with a small extended flagellum. In the midgut, the spheromastigotes elongate and further extend their flagella, differentiating them into epimastigotes [35]. The long-flagellated epimastigotes migrate to the hindgut and anchor to the perimicrovillar membranes of the hindgut intestine. This anchorage initiates the non-infective epimastigotes differentiation to infective metacyclic trypomastigotes which detach from the intestinal membranes and are migrated to the rectum where they are excreted in the feces and urine of the triatomine bug, infecting mammalian hosts [36].

### 2.2. Epidemiology of Trypanosomatids 

Trypanosomatids are a family of parasites causing infectious diseases categorized into trypanosomiasis and leishmaniasis. Trypanosomiasis is further subdivided into American and African diseases, also known as Chagas disease and African sleeping sickness, respectively. Leishmaniasis is also further classified based on its prevalent geographical location. Trypanosomiasis and leishmaniasis are neglected diseases widely observed in underdeveloped countries, mainly in Southern Asian countries, African countries, and South American countries [36]. Various factors influence trypanosomiasis and leishmaniasis species and their development, including carrier vectors, vertebrate hosts, biochemical enzyme patterns, parasite phylogeny, and geographical distribution [37,38]. Table 1 lists the main *Leishmania* and *Trypanosoma* species.

### 2.3. Clinical Complications and Conditions of Leishmaniasis and Chagas Disease

Leishmaniasis and Chagas disease cover a broad disease spectrum that affects humans and other mammals. Clinical manifestations and treatment options for leishmaniasis and Chagas disease are limited due to various factors, including the complexity of the parasitic species and subtypes. This makes it very difficult for doctors to determine the disease’s type and choose the optimal treatment plan [55].

#### 2.3.1. Leishmaniasis

##### Cutaneous Leishmaniasis (CL)

**Figure 2:**
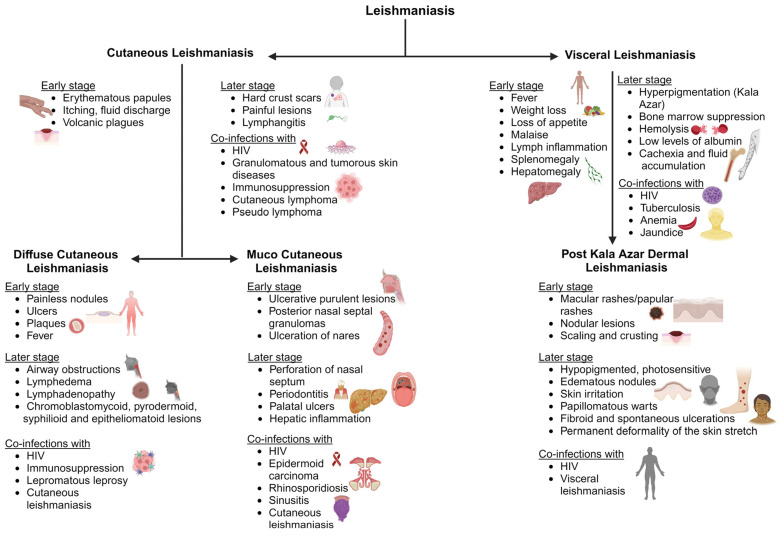
Clinical presentation of leishmaniasis. Clinical manifestations of individual leishmaniasis types during infection. It is characterized by early and later stages of infection and possible conditions. Infection is associated with other types of leishmania infections, complicating the patient’s condition. The figure also elaborates on the possible co-infections with leishmaniasis to provide the broader aspects of the infection. BioRender.com (accessed on 25 September 2023) was used to generate this Figure.

Cutaneous leishmaniasis (CL) is the most typical clinical symptom of *Leishmania* infection. Initial signs include single or multiple skin lesions localized to exposed body parts such as the face and may result in social stigma [56]. Numerous painless brownish erythematous papules with multiple nodules that resemble plague are seen after an incubation period of between two and seven months. It is followed by itchy and painful fluid discharge, warmth, swelling, and fever, which further develop into volcanic plagues associated with lymphangitis that differentiate from other ulcerative plagues (Figure 2). Bacterial and fungal infections are common on exposed cutaneous lesions, causing secondary infections [57]. There are findings of co-infection with diseases like helminthiasis, leprosy, Human African trypanosomiasis, and Chagas disease [58]. A few reports of leishmaniasis co-infection with immunosuppressed patients under Human immunodeficiency virus (HIV) treatment have also been observed among patients. There have also been granulomatous and tumorous skin diseases, including subcutaneous mycosis, deep mycosis, cutaneous lymphoma, pseudolymphoma, basal cell carcinoma, and squamous cell carcinoma, among patients (Figure 2) [59]. Due to these clinical complications, doctors cannot use a specific treatment regimen to treat the disease. 

##### Diffuse Cutaneous Leishmaniasis (DCL)

Diffuse cutaneous leishmaniasis (DCL) is widely observed in patients with a suppressed cell-mediated immune response against the invading parasite, also termed anergy [60]. DCL is characterized in patients with a low count of CD4 T-cells and immediately after antiretroviral drug therapy [61]. Several *Leishmania* (L.) species such as *L. aethiopica*, *L. major*, *L. amazonensis*, *L. mexicana*, and *L. braziliensis* cause DCL [62]. An initial symptom is erythematous lesions with a well-defined periphery that extend to mucocutaneous junctions including nodules, ulcers, and plaques [63]. As the lesions progress, they spread to the face, buttocks, and extremities and ulcerate the entire surface. The nodules across the nasopharynx and oropharynx cause airway obstructions and may be associated with lymphedema and lymphadenopathy, where the lesions are observed as sporotrichoid in morphology [64,65]. The lesions also resemble other chronic lesions [66]. DCL is associated with immunocompromised diseases such as acquired immunodeficiency syndrome (AIDS) and lepromatous leprosy [67]. These findings are a major clinical observation in defining *Leishmania* infection.

##### Mucocutaneous Leishmaniasis (ML)

The majority of mucosal/mucocutaneous leishmaniasis (ML) is transmitted by *L. braziliensis*, *L. amazonensis*, *L. panamensis*, *L. infantum*, and *L. guyanensis* [68]. It is initially painless, but ulcerative and purulent lesions develop. They continue as erythema, edema, posterior nasal septal granulomas, and ulceration of the nares [69,70]. It spreads to the oral cavity of the oropharynx and larynx. This causes perforation of the nasal septum, periodontitis, palatal ulcers, and gingivitis, damaging the vocal cartilage and resulting in speech abnormalities (Figure 2) [71]. Lymphedema leads to systemic infection causing fever and liver inflammation. Secondary complications include airway obstruction and fungal and bacterial infections [72]. The associated infections include epidermoid carcinoma, rhinosporidiosis or sinusitis, and AIDS, where a differential diagnosis is required (Figure 2) [69]. ML is associated with cross- and co-infections, which can worsen physical and psychological conditions.

##### Visceral Leishmaniasis (VL)

Visceral leishmaniasis (VL) is usually caused by *L. donovani* and *L. infantum*. These species are commonly found across North and East Africa, the Indian subcontinent, and Southern American nations. Infection is prevalent in all age groups but most prevalent in immune-suppressed patients and children [11]. The parasite incubates for about two to six months and leads to chronic infection lasting for several years. The initial clinical observations include fever, weight loss, loss of appetite, and a malaise that progresses to a few months and is followed by enlarged lymph nodes, splenomegaly, and hepatomegaly [72]. The disease is also called Kala Azar due to hyperpigmentation of the skin and visceral regions. These hyperpigmentation patches are observed as dark patches stretching the skin surface associated with abdominal congestion and pain as the infection continues. The patient also suffers from bone marrow suppression, hemolysis, low albumin levels, jaundice, thrombocytopenia, cachexia, fluid accumulation, and bleeding from the mucosal cavities [73]. This may be followed by hemophagocytic lymph cytosis and intravascular coagulation [74]. 

Infections of the oral mucosa, nasal, and gastrointestinal tracts are prone to bacterial and fungal contamination. This leads to sepsis and pneumonia which is fatal in children and geriatrics. It could cause abortion or congenital leishmaniasis in pregnant patients [75]. HIV infection, tuberculosis, anemia, and jaundice cases are frequently reported as co-infections with VL and increase VL mortality in southern Asian and African countries [76,77]. Post kala azar dermal leishmaniasis is also common after recovery from VL, which may lead to other medical complications.

##### Post Kala Azar Dermal Leishmaniasis (PKDL)

Post kala azar dermal leishmaniasis (PKDL) is mostly observed post VL treatment. It could be defined as the final stage of VL infection before complete recovery from the disease. It is mostly observed in Asian and African countries as VL is a concern in these regions [78]. African and Asian PKDL can be differentiated by macular rashes in Asian countries and papular rashes in African countries [79]. The onset of PKDL symptoms usually starts a few months post VL treatment. This mimics a chronic skin infection that lasts for three to four years if untreated. The initial clinical presentations include dispersed hyperpigmented maculopapular and nodular lesions on the face which spread to the chest and arms. These lesions cover the entire length of the skin with scaly skin and dense nodular skin rashes (Figure 2). The lesions or rashes may be ulcerative, erythematous, or keloidal [80]. The nodules and papules are hypopigmented, photosensitive, and edematous, with a succulent nature, which irritates the skin. They appear warty, papillomatous, and fibroid, with spontaneous ulcerations in the peritoneal area, chin, and parts of the face [81]. As the infection spreads to sensitive parts such as the tongue, groin, genitalia, and axilla, it can cause permanent deformity in the patient. Treatment approaches should be enhanced against infection and active surveillance should be carried out for a better understanding of how diseases spread.

#### 2.3.2. Chagas Disease

Clinical outcomes of *T. cruzi* infection are divided into two categories: acute and chronic stages. Before the infection progresses to the chronic stage, it incubates in the intermediate stage, which mediates chronic infection. We discuss each clinical complication and condition of Chagas disease in detail below.

##### Acute Chagas Disease

**Figure 3:**
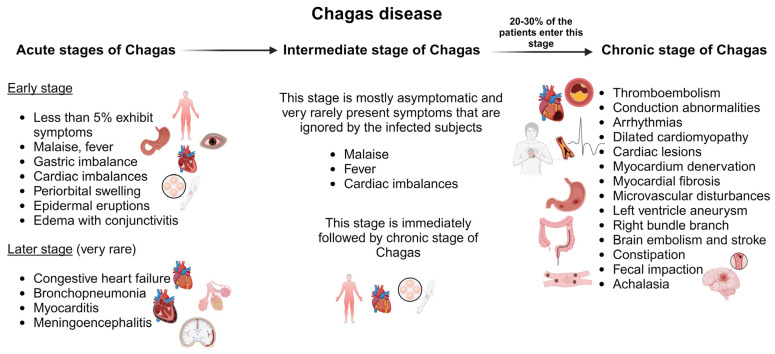
Diagram of a clinical representation of Chagas disease. Chagas disease has two stages or clinical phases: an acute and a chronic phase. Most infected people (between 70 and 80%) remain asymptomatic their entire lives, but in 20 to 30% of cases, the disease progresses to cause chronic symptoms. The infection is also associated with other diseases and infections, thus complicating the patient’s condition. BioRender.com (accessed on 25 September 2023) was used to generate this Figure.

The acute stage of Chagas disease is mostly asymptomatic and unnoticed by the patient. Symptoms are rarely observed after 30 days of incubation. During this stage, invasive trypomastigotes can be detected in fresh blood smears [82]. Less than 5% of patients in their acute stage of infection display symptoms such as malaise, fever, gastric imbalance, cardiac imbalances, inflammation at the inoculation site, periorbital swelling, epidermal eruptions, and edema with conjunctivitis indicating cutaneous infection (Figure 3) [83]. It might be accompanied by anemia, thrombocytopenia, hepatomegaly, and splenomegaly [84]. Death during this stage of infection is very rare and is primarily due to congestive heart failure, bronchopneumonia, myocarditis, or meningoencephalitis [85]. Children are more prone to death and are affected by myocarditis [86]. The acute stage persists for three–four months and then the infection enters the intermediate stage, which lasts for six–eight weeks.

##### Chronic Chagas Disease

The immediate phase after the acute stage is asymptomatic. Clinically, the patient has a normal electrocardiogram (ECG), gastrointestinal findings, and healthy physical condition but positive serological findings for infection. The intermediate phase of infection is followed by the chronic phase of infection, which is fatal [87]. Around 20–30% of patients with acute and intermediate infections progress to the chronic phase of Chagas disease, which can have highly symptomatic symptoms. This includes thromboembolism, conduction abnormalities, arrhythmias, and heart failure due to dilated cardiomyopathy (Figure 3) [82]. The severity of the disease depends on the duration, location, and nature of the cardiac lesions. The parasite resides in the cardiac tissues and causes immune activation in the myocardium. This causes denervation, myocardial fibrosis, microvascular disturbances, and myocardial injury [88,89]. Cardiomyopathies in Chagas disease manifest as ventricular remodeling, dysautonomia, and imbalances in cardiac perfusion [90,91]. Chronic Chagas cardiomyopathy is defined as a left ventricle aneurysm. This leads to an increase in left atrial pressure resulting in systolic dysfunction and reduced left ventricular filling [92]. The right bundle branch block is a common ECG found in chronic Chagas disease and is mostly found in association with an anterior fascicular block [84]. The chances of sudden death under this condition are very high due to ventricular tachyarrhythmia [93].

Damage to the brain and stroke are very common in Chagas disease due to hypoxia and reduced blood supply to the brain [84]. Brain embolism is a clinically recognized event associated with stroke in asymptomatic patients [82]. Other clinical manifestations include mega syndrome, which is a result of the denervation of tubular structures of the enteric nerves that modulate normal gastrointestinal function directed towards acute infection in the rectum, colon, and esophagus (aka oesophagus). In the colon and rectum, it leads to bowel dysmotility, constipation, and fecal impaction. In the esophagus, it causes achalasia. Also, it causes dysphagia, odynophagia, weight loss, idiopathic achalasia, regurgitation, cough, and chronic aspiration [83].

### 2.4. Pathways Involved in Leishmaniasis and Chagas Disease 

Trypanosomatids survival and differentiation involve various pathways. The parasite cannot synthesize all the metabolites needed for its survival, so it interferes with the host’s biochemical mechanisms to fulfill its nutritional needs. There are enzymes that resemble host intermediates and are salvaged into the parasite to create a safe environment, escape the immune system, and develop for their own purposes [94]. Some of the biochemical pathways affected by trypanosomatids infection are discussed below.

Sterols serve as a key metabolic element for *Leishmania* and *T. curzi* survival. Both these parasites increase ergosterol production by interfering with host sterol biosynthesis [94]. *Leishmania* and *T. curzi* parasites infest macrophagic sterol levels for parasitic differentiation, cellular shape, and division. This results in lowering cholesterol levels in macrophage plasma membranes [94,95]. In Chagas disease, cholesterol is employed in the endocytic vesicle and cytostome of amastigotes [96]. It leads to CD40 signaling pathway disturbances and alters Interleukin 12 (IL-12) production. It also increases arginase I expression, which inhibits NO synthesis and favors parasite survival and differentiation [97,98].

Both *Leishmania* and *T. curzi* are incapable of synthesizing the purines required for their differentiation and survival; hence, they salvage the purine molecules used by the host for their living [99]. Purine phosphoribosyl transferase assists *Leishmania* and *T. curzi* salvage purines from the phosphorus vacuole. It helps in catalyzing the formation of guanosine monophosphate and inosine monophosphate, which is required for parasite survival [100,101]. Typically, hypoxanthine–Guanine Phosphoribosyltransferase (HGPRT) and xanthine Phosphoribosyltransferase (XPRT) enzymes are involved in purine salvage [102]. Other enzymes such as adenine phosphoribosyl transferase (APRT), hypoxanthine–guanine–xanthine phosphoribosyl transferase (HGXPRT), adenosine kinase (AK), and nucleoside hydrolase (NH) were also identified to play a major role in acquiring purines from the host in cases of both *Leishmania* and *T. curzi* infections [103,104].

Cell surface proteins are anchored by glycosylphosphatidylinositol (GPI) molecules. GPI molecules, including Lipophosphoglycan (LPG) and Gylcoinositol phospholipids (GIPL), are synthesized by *Leishmania* and *T. curzi*, which help them anchor to macrophages and internalize them [105]. Most GPI anchors require trimannose backbones and dolichol–phosphate–mannose (DPM), which act as mannose donors [106]. These GPI molecules help host and parasite communication and immune invasion [107]. Mucins also play a role in anchoring parasite development. Trans-sialidase is one such mucin responsible for active anchorage and infectivity [108]. Both *Leishmania* and *T. curzi* contain mucin, but *T. curzi* contains more than *Leishmania* [109]. Molecules such as phosphatidylinositol, inositol phosphorylceramide, glycoproteins 63, glycoprotein 82, and glycoprotein 30 also assist in parasite anchorage and internalization of both *Leishmania* and *T. curzi* parasites. Also, these molecules facilitate the mobilization of intracellular calcium and the exocytosis of lysosomes, leading to a cascade of signaling between the host and the parasite for cellular invasion [110]. 

Folic acid and pteridines play a major role in *Leishmania* and *T. curzi* metabolic interventions [111]. Tetrahydrofolate is essential for purine, thymidylate, and pantothenate biosynthesis. Also, it is required for RNA protein formation and is involved in various signaling cascades [112]. Dihydrofolate reductase (DHFR) and Thymidine synthase (TS) act as bifunctional enzymes as they are both connected to N- and C-terminals, respectively, and separated by a linker peptide [113]. DHFR-TS helps protect dihydrofolate and drives cellular colocalization and parasite survival [114]. Pteridine reductase I (PTR-1) also plays a role in the conversion of biopterin to tetrahydrobiopterin when DHFR-TS inhibitors are used. PTR-I is activated when DHFR-TS is inhibited or malfunctioning and is responsible for parasite survival and a proper survival environment for their growth [115]. Trypanothione (TSH2) is a principal metabolite that protects *Leishmania* and *T. curzi* parasites from oxidative stress and is responsible for parasite differentiation. The formation of TSH2 is mediated by glutathionyl spermidine synthase (GSPS) and trypanothine synthase (TRYS). TSH2 is involved in the reduction in host glutathione and decreases macrophage oxidative stress during infection [116]. TSH2 is maintained at reduced levels as required by the parasites by trypanothione reductase (TR) [117]. TR shares 67% similarities among *Leishmania* species and 80% similarities among trypanosome species [118]. In *Leishmania*, TSH2 catalyzes the hydroperoxide reduction with the assistance of two proteins, namely tryparedoxin (TXN) and tryparedoxin-dependent peroxidase (TDPX). This reduces oxidative stress and enhances parasite survival rates [119]. TSH2 also possesses the properties of xenobiotics and endobiotic neutralization and enhances the reduction in ascorbate and iron–sulfur complexes [120].

N-(2-amino2-hyroxymethyl) lysine, also called hypusine, is found in two eukaryotic proteins, eukaryotic translation initiation factor (eIF)-5A1 and eIF5A2 [121]. eIF5A is responsible for cell cycle regulation, apoptosis, translation, elongation, and termination [122]. The post-translational modification leads to the binding of hypusine with lysine residues of eIF5A proteins by the transfer of 4-aminobutyl moiety from spermidine to the lysine chain, which is aided by the enzymes deoxyhypusine synthase (DHPS) and deoxyhypusine hydroxylase (DOHH), also known as hypusination [123]. Arginine serves as a precursor for eIF5A, which is essential in hypusine synthesis [124] and determines *Leishmania* and *T. curzi* infection rates. Arginine is translocated to parasites using amino acid permease 3 (AAP3) [125]. Arginine plays a role in the polyamine pathway. In *Leishmania,* the promastigotes accumulate arginine, utilize it in polyamine biosynthesis, and regulate arginine transport under conditions of arginine deprivation [126].

## 3. Drug Discovery Strategies and Insights into Current Therapeutics

### 3.1. Approaches to Drug Development against Leishmaniasis and Chagas Disease 

Drug discovery strategies for leishmaniasis and Chagas disease have evolved based on various approaches, such as structural biology, genetic engineering, molecular modeling, and high-content screening platforms [127,128]. Identification of functional molecular targets is crucial to target-based drug discovery [129]. Subsequently, molecules are crafted to disrupt specific targets crucial for parasite survival [16]. High-throughput, targeting-based drug screening assays were developed based on target identification from *T. cruzi* genome data [130]. In drug development, two different mechanisms are assumed for compound identification such as target-based drug discovery (TDD) or phenotype-based drug discovery (PDD) [16]. Identifying new drugs for inadequately treated medical conditions relies on TDD [129]. However, PDD involves evaluating diverse chemicals in contradiction to the pathogen’s phenotype in a biological system and animal model. For example, benzothiophene analogs emerged as novel agents from a phenotypic screen against GSK’s kinetoboxes [131,132]. However, the increased cost and high rates of failure of traditional drug discovery and development approaches have provoked the community to explore alternative approaches, such as computer-aided drug discovery which comprises structure-based, ligand-based, and system-based approaches to drug discovery [133,134]. 

Structure-based drug discovery (SBDD) is the use of 3D structures of molecular targets to increase ligand–receptor complementarity. It is mainly obtained by biophysical techniques and used in research for the determination of pharmacological targets [135]. For example, virtual screening approaches have recently been undertaken using imidazole–pyridine as a reference structure [14]. In addition, the identification of oxidosqualene cyclase as a novel molecular target in *Leishmania* could lead to promising programs to discover new agents for leishmaniasis treatment [38]. Finally, the ligand-based drug design (LBDD) approach also identifies key characteristics that contribute to biological activity while improving or identifying new chemotypes [136,137,138]. 

### 3.2. Current Drugs for Leishmaniasis and Chagas Disease

The chemotherapy currently used for leishmaniasis and Chagas disease includes a limited range of drugs that are used in combination or as monotherapy [55,139]. Currently, there is no vaccine available to prevent leishmaniasis or Chagas disease. Treatment plans vary depending on factors such as disease type, parasite species, and geographic location. Each year, thousands of compounds are tested for leishmaniasis and Chagas disease drug discovery. For the discovery of new treatment leads, high-throughput screening (HTS) campaigns have been conducted [140]. To identify new drug candidates for diseases like leishmaniasis and Chagas, the Drugs for Neglected Diseases Initiative (DNDi) is actively incorporating natural compounds and synthetic compounds into its portfolio. Several pharmaceutical companies collaborated with the DNDi in 2015 to launch the NTD Drug Discovery Booster [141]. As part of its ongoing efforts, the DNDi is initiating the ‘Chagas Hit-to-Lead’ project, which aims to identify promising leads in animal models of the disease, as well as develop innovative approach to discover possible drugs for treatment [141,142].

The majority of the mechanisms of action of drugs used today to treat leishmaniasis and Chagas disease are unknown. Further, many of them are ineffective, and their overuse is associated with a variety of emerging resistance patterns, as well as severe side effects [143,144]. Therefore, the development of novel drug discovery programs for anti-leishmanial and anti-trypanosomal compounds is essential [145,146]. The NTD Drug Discovery Booster (DDB) was launched in 2015 to avoid early-stage commercial obstacles between pharmaceutical participants, allowing the DNDi to search millions of unique compounds and use computational methods to refine the search iteratively in the hunt for new treatment leads [147]. Since its creation in 2015, the NTD-DDB has launched 45 iterations around 22 seed compounds, with 13 hit series released, of which 6 have progressed to in vivo proof-of-concept studies for Chagas disease, leishmaniasis, or both. Two compounds from one hit series have demonstrated efficacy in a leishmaniasis infection model: one was transitioned into lead optimization and is progressing to the pre-clinical stage. In 2017, more than 20 novel series were identified and are currently being processed [147].

#### 3.2.1. Leishmaniasis

**Figure 4:**
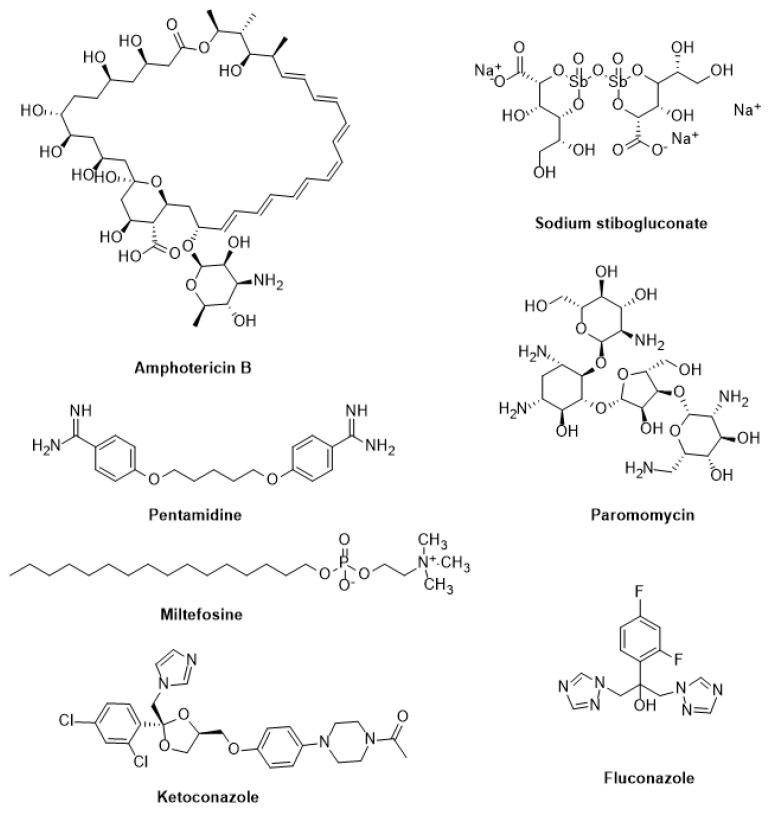
Chemical structures of commonly available leishmaniasis drugs.

Sodium stibogluconate and meglumine antimoniate have been used extensively to treat visceral, cutaneous, and mucocutaneous leishmaniasis for decades [148]. Yet, their parenteral therapy is long, the drugs are toxic, and resistance is on the rise. Therefore, other medications such as pentamidine, paromomycin, fluconazole, ketoconazole, miltefosine, and amphotericin B have been repurposed (Figure 4) [149]. Additionally, it has been noted that amphotericin B therapy does not result in a sterile cure and is linked to rising kala azar leishmaniasis rates, as well as the emergence of resistant parasites in clinical settings [150]. Although repositioning efforts have shown promise in discovering new treatments for this disease, the identification of novel therapeutic targets and the development of effective leishmanicidal drugs should be prioritized [151].

Miltefosine: Miltefosine (hexadecyl-phosphocholine) is the “only oral drug” available for leishmaniasis chemotherapy [152]. It is an amphipathic alkyl phosphocholine drug with a polar phosphocholine group and a long alkyl chain. Originally used to treat breast cancer, it has shown great promise as an anti-leishmanial agent since the 1980s, particularly in antimonials-resistant cases [153]. Miltefosine works primarily by (a) changing the composition of plasma membranes by inhibiting phospholipid and alkyl lipid metabolism [154], (b) causing programmed cell death by damaging mitochondria (depolarization) and obstructing cytochrome c oxidase [155], and (c) increasing nitric oxide synthetase 2 (iNOS2) expression in host macrophages, which produces nitric oxide (NO), which is toxic to parasite survival [154]. 

Amphotericin B: Also known as Amp B, this antifungal antibiotic exhibits a high affinity for the fungal membrane ergosterol and a low affinity for the host membrane cholesterol [156]. Amp B was identified in 1960 as a promising anti-leishmanial candidate due to its groundbreaking in vitro activity [157]. Following resistance to antimonials in India, Amp B became the first medication of choice to treat VL. By changing the membrane fluidity and creating macropores and micropores that leak essential components into the cells, it binds 24-substituted sterol (ergosterol) to the biosynthesis pathway and causes cell death [10]. Other processes that prevent the parasite from surviving include auto-oxidation and reactive oxygen species (ROS) [158].

Paromomycin: Paromomycin is an antibiotic isolated from *Streptomyces rimosus* and has a broad range of clinical applicability. It is very helpful against many Gram-positive and Gram-negative bacteria, as well as infections like giardiasis, amoebiasis, and cryptosporidiosis [159]. It is a member of the aminoglycoside class. Its qualities as an anti-leishmanial agent were identified in the 1960s, and it has since been demonstrated to be a successful treatment for both CL and VL [160]. As a result of binding to its 30S smaller subunit, paromomycin hinders protein synthesis machinery. Consequently, the ribosome cannot be recycled due to this binding, which further halts protein synthesis. The membrane potential can also be disrupted by lipid metabolism and membrane fluidity changes [161]. 

Pentamidine: Pentamidine has been used as a second-line treatment for VL, CL, and DCL for over 40 years. Despite its reintroduction in the 1990s after clinical trials for New World CL [162], pentamidine is barely used as an anti-leishmanial drug. Pentamidine’s anti-leishmanial mechanisms of action remain unclear. However, they may affect the mitochondrial inner membrane potential, inhibit polyamine biosynthesis, and bind to minor grooves in DNA. Clones of *L. donovani* and *L. amazonensis* that are resistant to pentamidine exhibit increased efflux and reduced uptake by 18 and 75 folds, respectively [163]. Additional information suggests that pentamidine accumulation in the leishmania mitochondria is significant. However, specific transporters for pentamidine uptake have been identified and may play a role in resistance. In comparison to resistant cells, wild-type promastigotes accumulate more pentamidine in the mitochondria [163,164]. 

#### 3.2.2. Chagas Disease

**Figure 5:**
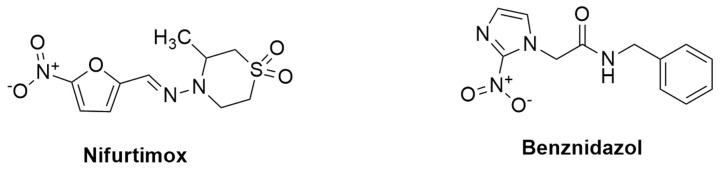
The chemical structures of currently used drugs against Chagas disease.

Nifurtimox (NFX) and Benznidazole (BZN) are the two main drugs used to treat Chagas disease, and both were developed more than 50 years ago (Figure 5). The treatment is lengthy (60–90 days) and ineffective for chronic patients, in addition to having a toxic effect and the potential for evolving resistance [165,166,167]. Although the exact mechanism underlying Benznidazole’s activity is unknown, it is activated by NADH-dependent trypanosomal reductases and produces reductive metabolites that are thought to have several negative consequences, including DNA damage and protein synthesis inhibition [168]. The FDA approved benznidazole for the treatment of Chagas disease in children aged 2 to 12 years old in 2017. It was the first medicine licensed in the United States for Chagas disease. A new drug that is both safe and effective for Chagas disease’s acute and chronic stages is required. However, several factors impediment the development of new candidate drugs, including the lack of biomarkers for the two stages of the disease and for evaluating treatment success, as well as the genetic diversity of *T. cruzi* strains [169,170].

Nifurtimox and Benznidazole: Nifurtimox and benznidazole are the current drugs used to treat Chagas disease. Benznidazole’s mechanism of action is a prodrug that is metabolized by the parasite to form free radicals that cause oxidative damage to the parasite’s macromolecules, ultimately leading to death. These free radicals are thought to harm the parasite’s DNA, proteins, and lipids [171]. Nifurtimox’s mechanism of action is unknown, but it is thought to be related to its metabolism via partial reduction to chemically reactive radicals that produce oxygen radicals such as superoxide, hydroperoxide, and hydroxyl. These radicals cause oxidative stress in the parasite, which ultimately leads to death [172]. Table 2 describes the summarized mechanism of action. 

Current treatments for leishmaniasis and Chagas disease have several problems. Most of them have side effects, treatment failures, and relapses caused by drug resistance. These anti-trypanosomal and anti-leishmanial medications also have drawbacks that exclude their use in specific population cohorts or under specific conditions. For example, several of them are teratogenic and inappropriate for pregnant women and newborns. Another concern is the poor oral bioavailability of most of these drugs [173]. Chagas disease and leishmaniasis treatments also differ depending on the endemic location. Table 2 summarizes the WHO-recommended treatment regimens for major Chagas disease and leishmaniasis endemic sites. Treatment alternatives are generally insufficient, and new medications are desperately needed. Most drugs used to treat Chagas disease and leishmaniasis are on the 19th edition of the WHO Model List of Essential Medicines, including pentavalent antimonials (SbV), miltefosine, amphotericin B deoxycolate or formulated in liposomal formulations, paromomycin, and pentamidine (Figure 4 and Figure 5). 

**Table 2:** Current pharmacological treatments against Chagas disease and leishmaniasis.

Disease	Drug	Route	Mechanism of Action	Disadvantages	Toxicity	References
Leishmaniasis	Miltefosine	Oral 5–100 mg/kg/day for 28 days	An anti-apoptotic agent targeting lipid metabolism, mitochondria, and immune function	Need allometric administration in children	Gastrointestinal complications, teratogenic	[174,175,176]
Leishmaniasis	Paromomycin	Parenteral (im) 15 mg/kg/day for 21 days	There is little knowledge about this. Several studies show that cationic paromomycin binds negatively charged glycocalyxes and lipophosphoglycans found on the surfaces of leishmania promastigotes, a major component of leishmania promastigotes	Poor results against African VL as monotherapy	Pain in the injection site, hepatotoxicity	[177,178,179]
Leishmaniasis	Pentamidine	Slow infusion (iv) 4 mg/kg monthly for 12 months	Not known; drug entry through polyamine/arginine transporters	Multiple adverse effects	Insulin-dependent diabetes, myocarditis, nephrotoxicity	[180]
Leishmaniasis	SbV—paromomycin combination	Parenteral (im) SbV 20 mg/kg/day + paromomycin for 17 days	Currently, there are two models of Sb(V) activity: the prodrug model of conversion to toxic Sb(III) and the intrinsic Sb(V) activity based on complex formation with ribose/inhibition of type I DNA topoisomerase.	Require hospitalization	Problems regarding SbV administration	[174,181]
Leishmaniasis	Amphotericine B deoxycholate	Slow infusion (iv) 1 mg/kg/day for 30 days	Channel/pore formation on interaction with membrane sterol	Require hospitalization	Nephrotoxicity	[182,183]
Leishmaniasis	SbV—based drugs	Parenteral (im) 20 mg/kg/day for 28–30 days	Prodrug model conversion of Sb(V) to toxic Sb(III) and intrinsic Sb(V) activity: by inhibiting type I DNA topoisomerase/complex formation with ribose	Drug resistance in Bihar (India), PKDL	Pain in the injection site, cardiotoxicity, pancreatitis	[184,185]
Leishmaniasis	AmBisome	Slow infusion (iv) 10 mg/kg single dose	Channel/pore formation on interaction with membrane sterol	Costly, chemically unstable	Fever during infusion, back pain, nephrotoxicity	[186,187]
Chagas disease	Benznidazole (BZL)	Given for 60 days on daily basis at 5–7 mg/kg, and 10 mg/kg for adults and children, respectively	Inhibits the synthesis of DNA, RNA, and proteins within the *T. cruzi* parasite	Low solubility, toxic, and several side effects	Low bioavailability and drug effectiveness, chronic effects	[86,188,189,190]
Chagas disease	Nifurtimox (NFX)	8–10 mg/kg daily in three divided doses for adults, and 15–20 mg/kg daily in four divided doses for children during 60 to 90 days	Metabolism via partial reduction to chemically reactive radicals that cause production of toxic reduced products of oxygen	Toxic and have side effect, causes gastrointestinal, maladies (nausea, vomiting, abdominal pain) effects	Have higher toxicity and adverse effect than BZL, and it affects the pancreases and heart via increasing of oxidative stress	[191,192,193]

Abbreviations: im—intramuscular, iv—intravenous.

**Figure 6:**
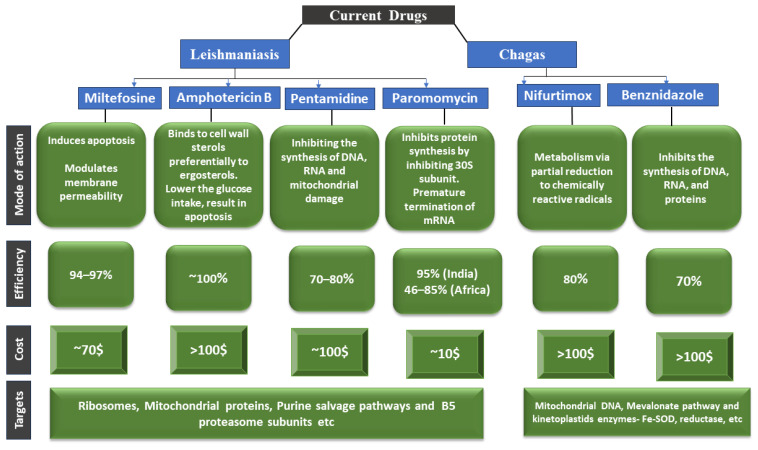
The mode of action, biochemical characterization, and potency of current drugs in leishmania and Chagas infection against the parasites.

The current drugs for leishmaniasis and Chagas disease treatment have limitations and challenges. Drugs currently available to treat Chagas disease and leishmaniasis differ in their mode of action, their efficacy, their cost, and their targets [194]. Paromomycin (injectable, long treatment, region-dependent efficacy), miltefosine (cost, teratogenicity, long treatment), and liposomal amphotericin B (cost, hospitalization, region-dependent efficacy) are three examples of drugs. There are only a few validated drug targets known against these parasitic diseases, and many drugs used to treat them have serious side effects and limited efficacy [195]. A severe lack of drug targets that have been rigorously validated genetically and chemically has hampered the development of better and safer drugs to treat these diseases [196,197]. The mode of action of some drugs used to treat these diseases is known [197]. Figure 6 depicts the mode of action, biochemical characterization, and potency correlation of the current drugs reported in the literature to treat Chagas disease and leishmaniasis.

## 4. Novel Strategies for Leishmaniasis and Chagas Disease Treatments

There is an urgent need to discover and develop compounds effective against leishmaniasis and Chagas disease, which demonstrate high bioactivity, low toxicity, and an acceptable cost and administration profile [16,198]. In the past two decades, numerous assays have been designed for use in screening programs targeting the various lifecycle stages of *Leishmania* and *Trypanosoma* parasites. As soon as active compounds are developed against the intracellular amastigote form of the parasite, they are tested in vivo, following a pharmacokinetic and pharmacodynamic assessment [140]. Barriers to the development of new drugs include the lack of well-characterized and validated targets, the absence of diagnostic biomarkers resulting in diagnostic failures, the variety of parasite strains, issues with the standardization of methodologies, such as various in vitro assays, different host cell culture lines, and issues with the translation process [141]. Further, the treatment of these deadly diseases is still challenging due to limited drug regimens, resistance, toxicities, co-infection cases, and low investments in new drug discovery/development [199,200]. Various strategies must be explored and implemented to combat these NTDs and overcome conventional drugs’ drawbacks. The following section summarizes recent developments and upcoming insights into traditional anti-trypanosomal and anti-leishmanial therapies. 

### 4.1. Host-Directed Therapy (HDT)

Host-directed therapy (HDT) aims to achieve a clinical cure by manipulating the host immune system rather than using drugs to treat the parasite [201]. HDT uses various molecules (e.g., repurposed drugs, immuno-modulators, synthetic nucleic acids, cytokines, cellular therapy, and micronutrients) that can boost the immune function of the host body or control the inflammatory response. Both have a long-lasting healing effect and lower mortality and morbidity [202]. A better understanding of the immune response to *Leishmania* has revealed the crucial fact that infection depends on the successful downregulation of the T-helper cell type 1 (Th1) immune response and the development of the Th2 responses [203]. Since the immune response profile in Chagas disease is similar to that in *Leishmania* [204], host-directed therapies relevant to *Leishmania* might also benefit patients with *Trypanosome* infection [204,205].

For example, simvastatin inhibits the pathway that produces cholesterol and causes macrophages to mature their phagosomes, increasing parasite clearance [206]. Similarly, lovastatin, which is chemically similar to simvastatin, is essential for lowering parasite internalization and infectivity in the J774A.1 macrophage cell line due to cholesterol depletion [207]. Eugenol (*Syzygium aromaticum*) and its derivatives have been hailed as promising treatments for inducing immunostimulatory responses that benefit the host. Eugenol derivatives inhibit leishmania promastigotes and amastigotes [208]. In conclusion, immuno-modulatory compounds may prove valuable candidates for treating leishmaniasis and Chagas disease. Some drugs/compounds identified through HDT are listed in Table 3. 

### 4.2. Multi-Drug or Combination Therapy 

The primary goals of the combinational or multi-drug approach are to shorten the treatment period, lower the relapse rate, reduce dosage, avoid fatal side effects, and combat drug resistance problems [174]. HIV, malaria, and tuberculosis have all been successfully treated using this approach [219,220]. Itraconazole, ezetimibe, and miltefosine were some of the drugs used in a multi-drug therapy approach to evaluate anti-leishmanial efficacy against the VL model of infection in BALB/c mice [203]. Due to its capacity to maximize the efficacy of currently available medications while lowering dosage requirements and treatment times, the combination therapy approach has recently seen significant use in the treatment of leishmaniasis and Chagas disease. A wide range of trustworthy combinations to treat leishmaniasis is possible. In the search for the most effective leishmaniasis treatment regimen, novel drug combinations can now be further investigated.

### 4.3. Drug Repurposing

Drug repurposing (aka reprofiling, drug repositioning, or re-tasking) is an approach that identifies novel applications for clinically approved drugs originally developed for another medical treatment [221]. This method is associated with a shorter drug development timeline and lower investment costs because the drugs have already undergone clinical studies [222]. Numerous medications have been repurposed for leishmaniasis and have shown promising results, such as Amp B, miltefosine, and azoles (fluconazole, itraconazole, and posaconazole) [223]. Numerous antidepressants, including Ketanserin, imipramine, clomipramine, nitroimipramine, sertraline, etc., have shown promising results when used as repurposed anti-leishmanial medications [223,224]. A recent study using transmission electron microscopy and metabolomics platforms identified the mechanism of sertraline’s leishmanicidal effect on *Leishmania* parasites [225]. Some of the drugs/compounds identified through drug repurposing are presented in Table 4. 

### 4.4. Promising Natural Products 

The Drug Discovery Research Program and the Tropical Diseases Program of the TDR (Tropical Disease Research) WHO intend to make plant pharmacology a priority. An article with a complete list of plants and natural products that demonstrated anti-leishmanial and anti-trypanosomal activities was published in 2015 [234]. Several studies have been conducted since then that demonstrate the pursuit of novel products derived from microbial or marine sources. For example, a glycoprotein isolated from the sponge *Pachymatisma johnstonii* showed strong in vitro activity against *L. donovani*, *L. braziliensis*, and *L. Mexicana* [235]. Essential oil’s anti-leishmanial activity has also been studied [236]. Advanced research has also evaluated additional potential substances isolated from natural sources that showed anti-leishmanial and anti-trypanosomal activities [196].

Valli et al. report on Cytochalasine A-D-based compounds that are similar to benznidazole or miltefosine in treating intracellular *T. cruzi* and leishmania that are comparable to the standard drugs of benznidazole and miltefosine [237]. Several recent studies concluded that natural products have better or comparable potency to conventional drugs in treating leishmaniasis and Chagas disease [238,239]. For example, Cortes et al. list natural products extracted from Old World flora that are warranted for leishmanial treatments [240]. Another study demonstrated that natural products could serve as effective chemotherapeutic agents against parasites by targeting mitochondrial homeostasis. The research revealed that these natural compounds can induce parasite death by impacting their mitochondrial function [241]. In conclusion, natural products have a bright future in drug design and research. 

### 4.5. Nanotechnology

Drug delivery and drug design can use nanomedicine, a branch of science that deals with particles at the nanoscale [242]. Low permeability, insolubility, painful injections, prolonged hospitalization, and unfavorable side effects are just a few of the limitations of the conventional delivery system. In the search for the most effective leishmaniasis treatment, researchers have also used nanoparticles (NPs). NPs are employed in drug delivery due to their biocompatibility, improved drug solubility, on-target drug delivery to the target organ, immuno-compatibility, and increased potential for a variety of administration routes [243]. Target-mediated drug delivery systems use metallic, inorganic, organic, and polymeric nanomaterials, such as carbon nanotubes, dendrimers, liposomes, and micelles [244]. Since phagocytic cells, particularly macrophages, are the target organs in leishmaniasis, liposomal derivatives and polymeric NPs are used for drug delivery. Higher ROS production, an increase in immuno-modulatory response, DNA damage, disruption of mitochondrial membrane potential and electron transport chain, and inhibition of trypanothione reductase enzyme vital for the *Leishmania* parasite’s anti-oxidation process are just a few of the mechanisms that NP-encapsulated drugs/molecules use to kill *Leishmania* parasites [245]. Although there are countless benefits, nanoscience’s use in practical life is limited. This is because it can sometimes have a significant adverse effect on the body due to its biological sensitivity [246]. Preparation and manipulation require specialized and engineered products, making it a cost-intensive task unsuitable for NTDs like leishmaniasis [247]. Some of the drugs/compounds identified through nanotechnology are listed in Table 5. 

### 4.6. Nano Vaccines

As a novel approach to vaccination, nanovaccines cause both humoral and cell-mediated immune responses, providing better treatment for several diseases, including leishmaniasis [255]. A chitosan nanoparticle-loaded recombinant superoxide dismutase (SODB1) vaccine was developed in 2011 using the ionotropic gelation method and evaluated on BALB/c mice. In both the single and triple doses of SODB1 nanoparticles, IgG2a and IgG2a/IgG1 were significantly higher than in the other groups [256]. A second study examined the efficacy of chimeric peptides containing HLA-restricted epitopes from three immunogenic *L. infantum* proteins in poly (lactic coglycolic acid) acid nanoparticles with or without monophosphoryl lipid A (MPLA) or surface modification. The nanovaccine activated non-producing CD8+ T cells specific for peptides and induced dendritic cell maturation [257]. Additional studies used synthetic peptide-based nanovaccines along with an MPLA adjuvant co-encapsulated in PLGA nanoparticles. The results demonstrated a strong spleen lymphoproliferative response and high levels of IL-2, interferon gamma (IFN-γ), and tumor necrosis factor α (TNF-α) versus low IL-4 and IL-10 secretion [258,259]. A 2019 study developed a process to prepare lipidic NPs loaded with plasmid pVAX1-NH36 for application as a leishmaniasis nanovaccine [236].

Developing novel therapeutics and substitutes for existing ones is one approach to treating leishmaniasis and Chagas disease [195]. As a means of treating these illnesses, researchers are creating innovative therapeutics and screening techniques to find new compounds/chemical diversity [195,260]. We described above some of the new treatment approaches for Chagas disease and leishmaniasis. As possible hits and leads, a large variety of compounds from several families have been found; some are even undergoing clinical testing [10]. Table 6 lists several candidate drugs that are being considered, including inhibitors that affect the metabolism of thiols, sterols, glycolytic processes, folate, and trypanothione. 

## 5. Peptide Targeting for Leishmaniasis and Chagas Disease Therapies

### 5.1. Peptide Therapies

Peptides are short chains of amino acids that play critical roles in human physiology. They were first used in medicine in the first half of the 20th century. Therapeutic peptides are exceptional pharmaceutical tools with a molecular weight of 500–5000 Da [270]. As technology advances, peptide drug discovery has become established. This includes drug design, peptide drug discovery, peptide synthesis, structural modification, and the association between these developments and peptide bioactivities [271,272]. Peptides have unique intrinsic characteristics which are produced and modified through chemical and biochemical methods associated with novel design and delivery strategies. Currently, in addition to many preclinical studies, more than 170 peptides are actively in clinical development [270,273] and sales total more than USD 70 billion [274]. Compared with biologics, therapeutic peptides show less immunogenicity and have lower production costs [275,276]. Compared to small molecules, peptides typically demonstrate higher potency and selectivity for their targets [277,278].

Therapeutic peptides have two basic limitations: low membrane permeability and poor in vivo stability. Yet, peptides as drugs have many advantages including high potency, high selectivity, and low toxicity. Thus, efforts using several models confirmed the effects of several peptides on various parasites (Table 7) [272,279,280].

### 5.2. Anti-Microbial Peptides

An anti-microbial peptide (AMP) is a small cationic molecule, amphipathic in structure, which has a variety of activities against viruses, bacteria, fungi, and parasites [289,290]. AMPs are more effective than conventional antibiotics, with a low bactericidal concentration and a quick ability to kill germs. They can even effectively combat bacteria strains resistant to conventional antibiotics [291]. Furthermore, AMPs can also overcome drug resistance in bacteria. Some of them have biological activities, such as anti-parasitic, anti-virus, and anti-fungal properties [291,292]. Current research efforts are focused on developing AMPs for therapeutic applications against NTDs, including leprosy, trachoma, African trypanosomiasis, Chagas disease, and leishmaniasis [292,293]. AMPs are effective against a variety of NTDs, including African trypanosomes, leishmaniasis, and Chagas disease. Research into the development of new anti-microbial agents has been driven by the increase in resistance to traditional antibiotics and emerging infectious diseases. Due to their direct anti-microbial killing activity and significant role in innate immunity, AMPs represent a promising alternative to existing antibiotics in the treatment and prevention of microbial infections [292]. To date, more than 2000 AMPs have been discovered, and many exhibit broad-spectrum anti-bacterial, anti-viral, and anti-parasitic activity [294]. Research on the potential application of AMPs’ structural and natural analogs in the fight against NTDs is ongoing [292].

The primary mechanism by Ih AIPs inhibit parasItic infection is by binding to and rupturing the plasma membrane of the parasites [294,295]. Most of these peptides are cationic–amphipathic, exhibiting two main mechanisms of action: direct lysis and modulating the immune system [289]. In addition to depolarizing membranes, disrupting plasma membrane permeability, and causing programmed cell death, these peptides also have major microbicidal effects. As a result, AMPs play a crucial role in the production of cytokines which modulate immune responses in the host [296,297]. Researchers working on parasites pay a lot of attention to AMPs, which are also called host-defense peptides [293]. Murine cathelicidin or cathelin-derived AMP was evaluated against *L. major* infection. Compared to control cells, J774A.1 cells expressed more cathelin-derived AMP in vitro. Furthermore, cathelin-derived AMP expression was upregulated at dermal inoculation sites in *L. major*-infected BALB/c mice, unlike the control group [203,297]. Additionally, protein assays showed that cathelin-related AMP levels increased in infected macrophages and mice models, suggesting that the immune response against *L. major* may be mediated by cathelin-related AMPs. A study conducted by Zahedifard et al. evaluated the leishmanicidal potential of Brevinin 2R (belonging to the class of defensins) alone or in combination with lauric acid against *L. major* parasites and found that mice treated with Brevinin 2R exhibited decreased parasite load in the lymph nodes [203,298].

Since AMPs have excellent leishmanicidal and immunomodulatory properties, they can serve as leads during drug discovery pipelines and vaccine design for leishmaniasis and Chagas disease. In general, AMPs receive more attention due to different factors such as antibiotic-resistant microorganisms [299]. While AMP mechanisms remain unclear, many AMPs cause swelling in the promastigote membrane, which leads to loss of integrity and substantial cell death [208]. The development of AMPs for therapeutic applications against NTDs faces several challenges, such as natural AMP lability, susceptibility to protease, pH changes, and potential toxicity [289,294]. The advantages of AMPs over conventional antibiotics include a slower emergence of resistance, broad-spectrum activity, and efficacy against drug-resistant Gram-positive organisms [291,300]. Despite these obstacles, AMPs have demonstrated promise for treating drug-resistant bacteria and infectious diseases, including non-therapeutic diseases like leishmaniasis and Chagas disease. Even though there are challenges to overcome, there are potential advantages in treating NTDs and AMPs represent a promising field for research and development.

#### 5.2.1. Anti-Microbial Peptides against Chagas Disease and Leishmaniasis

K777, a vinyl sulfone cysteine protease inhibitor, is considered a highly powerful and well-recognized cysteine peptidase inhibitor. It inhibits cruzain (aka Cruzipain), a key protease required for *T. cruzi* survival. It has not promoted a parasitological cure but did significantly reduce parasite-induced heart damage in vivo [301]. Over the past few years, several AMPs have been evaluated for their effects on *T. cruzi*, and apidaecin, magainin II, melittin, and cecropin A have been identified as potential candidates for Chagas disease as they kill *T. cruzi* in low concentrations [302].

AMPs are an essential protection mechanism and part of vertebrates’ native immunity. These peptides damage the protozoan’s cell membrane, affecting membrane integrity and producing lethal pores [303]. Over 2,000 AMPs have been identified from a variety of organisms, including bacteria, insects, plants, amphibians, birds, reptiles, and mammals, including humans. Some of these AMPs demonstrate leishmanicidal activity, such as halictine-2 from the poison of eusocial honey-bees [304]. Attacin, cecropin, and defensins from *lutzomyia longipalpis* respond to *Leishmania* infection [305], and Dragomide E., a linear lipopeptide isolated from the cyanobacteria *Lyngbya majuscula*, demonstrates anti-leishmanial activity against *L. donovani* promastigotes. The LZ1 peptide, derived from Snake cathelicidin, is an artificially designed and synthesized active polypeptide demonstrating high anti-microbial activity. It inhibits ATP production in parasite-infected erythrocytes [306]. Finally, Phylloseptin-1, a cationic peptide from the skin secretion of *Phyllomedusa azurea*, demonstrates high anti-parasitic activity and prevents cross-resistance because of its distinctive chemical structure [307].

**Figure 7:**
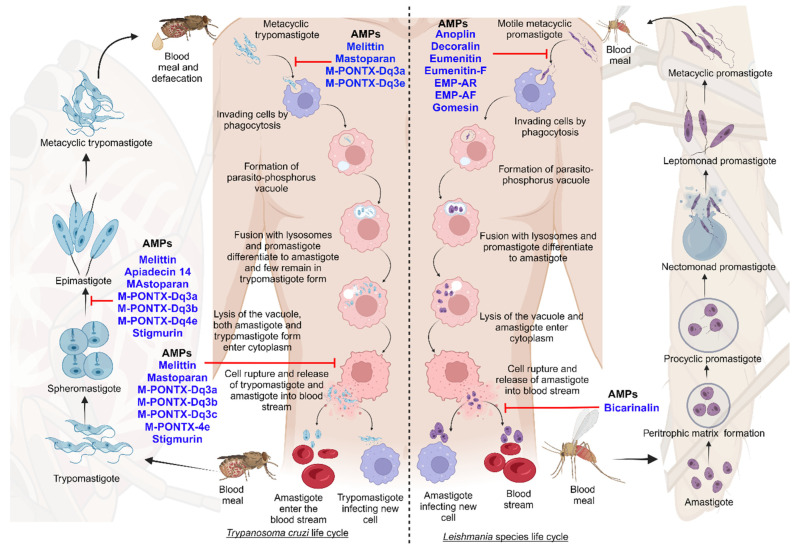
Schematic representations of *T. cruzi* and *Leishmania* life cycle-associated anti-microbial peptides (AMPs) against each specific developmental form of the parasite. The figure elaborates on different AMPs acting on parasitic differentiation stages in both host and vector. This provides a detailed view of the AMPs’ treatment regimen. BioRender.com (accessed on 25 September 2023) was used to generate this Figure.

The pharmaceutical industry recognizes that peptide-based drugs are a significant class of therapeutic agents. Anti-protozoal therapeutics can be derived from insects, bacteria, marine organisms, and amphibians [296]. In terms of parasite propagation, there are two stages in which the parasites can be investigated: the diagnostic stage (amastigotes multiply in cells) and the infectious stage (the promastigotes are injected into the blood) [157]. Currently, there are no specific details or clear evidence regarding the stages of clinical trials of AMPs against leishmaniasis and Chagas disease. Additionally, the pipelines for drugs to treat Chagas disease, leishmaniasis of the skin, mucocutaneous leishmaniasis, and PKDL are poor. New drugs are urgently needed to treat these diseases [308]. To achieve the WHO road map for neglected tropical diseases, new drugs are needed for leishmaniasis and Chagas disease [309]. In the case of tropical diseases, there are some studies of clinical trials investigating anti-microbial activities against bacteria [310,311]. The next figure describes some of the AMPs that have been identified and their functions against the different developmental forms of parasites (Figure 7) [303].

In general, several AMPs have shown promising activity against Chagas disease and leishmaniasis. Oxaborole DNDI-6148 has been nominated as a potential candidate for Chagas disease and is currently undergoing phase I clinical trials [139]. However, there is little progress in the pipeline for Chagas disease treatment. Other reports indicate that the acylated synthetic anti-microbial peptide Oct-CA (1-7)M (2-9) was used to treat leishmaniasis in dogs, but it is unclear whether it is currently in clinical trials [289]. Overall, several AMPs have demonstrated anti-trypanosomal and anti-leishmanial activity, and they are promising candidates for clinical trials.

#### 5.2.2. Structural Analysis of Selected Anti-Microbial Peptide Profiles against Leishmaniasis and Chagas Disease

AMPs are significant and essential defense mechanisms and highly active against various pathogens. AMPs are a component of the immune systems of vertebrates and invertebrates [303]. Most natural AMPs are short (10 to 50 amino acids) and have positive net charges (+2 to +11), with a significant portion of hydrophobic residues [312]. Due to their anti-parasitic properties, AMPs are used as pharmaceutical leads [313]. Positively charged residues on the polar face and net charge become essential for both anti-microbial and hemolytic activities [314,315,316,317]. Table 8 below summarizes the net charge and activities of selected AMPs for leishmaniasis and Chagas disease.

AMPs’ amino acid composition determines their charging properties and selective actions [329,330]. The cationic charge in their structures has a strong correlation with anti-microbial activity, and it is responsible for the initial electrostatic interaction between peptides and the anionic microbial surface through electrostatic interactions. The hydrophobic residues enable the AMPs to further insert into the membrane bilayer [331]. Positive-charge peptides are usually highly alkali at the N-terminal ends and rich in basic amino acids, while they are neutral hydrophobicity at their C-terminal ends [332]. Modification of AMPs is intended to increase their stability and efficacy while decreasing their cytotoxicity and untargeted side effects [333]. Peptides with higher net positive charges can be considered anti-microbial potential inhibitors. On the other hand, the hydrophobicity of those selected AMPs usually does not increase or decrease with net positive charges. There is an optimum hydrophobicity window for high anti-microbial activity. The decrease in anti-microbial activities at high peptide hydrophobicity can be explained by the strong peptide self-association, which prevents the peptide from passing through the cell wall in prokaryotic cells [334].

### 5.3. Protein–Protein Interactions as Drug Targets in Leishmaniasis and Chagas Disease

The human proteome contains approximately 30,000 proteins and significantly more protein–protein interactions (PPIs) that play critical roles in biological processes. Their dysregulation results in the onset and progression of a variety of diseases. PPIs thus represent a treasure trove of disease-modifying drug targets [335]. However, targeting these is difficult when converting drug-like small molecules into therapeutics. When targeting PPIs, it is critical to strike a balance between the interacting proteins to elicit a therapeutic effect while avoiding a significant adverse effect [336,337]. Researchers have reported that PPIs as a drug discovery strategy target a variety of diseases [337]. However, there is limited information about PPIs for leishmaniasis and Chagas disease. The proteins tryparedoxin peroxidase (PDB ID: 3TUE) [338], trypanothione reductase: (PDB ID: 5EBK) [339], and pyruvate kinase (PDB ID: 3PP7) [340] have recently been identified as structural macromolecules that play a variety of roles in the *Leishmania* parasite [4]. It is worth considering the importance of these proteins in the context of *Leishmania*, as well as their potential implications for future research into the parasite’s metabolism and pathogenicity. Therefore, PPIs offer the potential for further research into drug discovery for leishmaniasis and Chagas disease.

## 6. Conclusions and Future Directions

Leishmaniasis and Chagas disease are NTDs and global burdens. As a result of recurrent failures in leishmaniasis and Chagas disease diagnosis, chemotherapy is delayed and, eventually, this results in death. Despite the efforts of many research groups, leishmaniasis and Chagas disease remain without an effective solution. Challenges regarding leishmaniasis and Chagas disease include accurate and timely disease diagnosis, which is crucial in identifying asymptomatic patients and co-infected cases. However, recent diagnostic procedures such as advanced molecular techniques, proteomics, and nano-diagnosis may improve the prognosis of these diseases. Other key challenges are related to currently available treatments, including drug resistance and toxicities. These may be overcome by less traditional approaches, such as multi-drug and host-directed therapies, which have yielded promising results in recent studies. In addition, clinical trials with innovative treatment strategies should be performed to explore the impact of novel approaches on improving treatment efficacy, decreasing side effects, and lowering treatment costs. The review discusses the advancement of novel treatment strategies, featuring peptides as potential therapeutics against NTDs. Since peptides have specific actions and reduced toxicity, they are promising as new drug discovery targets and for development. It is evident that these targeted compounds are becoming increasingly popular within the pharmaceutical industry and hopefully they will greatly improve the health of many people suffering in the world’s poorest countries.

Existing anti-parasitic drugs for leishmaniasis and Chagas disease, in use for decades, are effective but pose significant concerns due to adverse effects and drug resistance. Drugs like pentamidine and amphotericin can lead to hospitalization and face resistance issues. High costs limit amphotericin’s accessibility. Miltefosine, that was originally developed as an anti-cancer drug and later was found to be useful for leishmaniasis, may cause teratogenicity and resistance. Chagas treatments, benznidazole and nifurtimox, exhibit adverse effects. Combination therapy faces limitations due to resistance and toxicities. This has prompted exploration of new strategies like host-directed therapy, nanovaccines, and natural extracts, showing promise with better stability and reduced toxicity. Vaccines against these diseases prove effective. Peptide-based therapies, despite stability and permeability challenges, offer low toxicity and resistance, making them potential candidates for leishmaniasis and Chagas treatment, exhibiting high efficacy at the infection site. Overall, peptides, vaccines, and novel approaches present alternatives with lower resistance, toxicity, and higher efficacy against parasitic infections.

## Figures and Tables

**Table 1:** Geographical and species distribution of leishmaniasis and Chagas disease.

No	Diseases	Species	Types	Geographic Distributions	References
1	Leishmaniasis	*Leishmania* (L.) *Aethiopica*	Cutaneous and mucocutaneous leishmaniasis	Ethiopia, Kenya	[39]
2		*L. Tropica*	Visceral and cutaneous leishmaniasis	Eastern and Northern India, Central Asia, East and South Africa, Middle East	[39]
3		*L. Amazonensis*	Cutaneous and mucocutaneous leishmaniasis	Brazil, Bolivia, Venezuela	[40]
4		*L. Infantum*	Visceral and cutaneous leishmaniasis	Mexico, Brazil, Bolivia, Venezuela, Northern Africa, Middle East, Mediterranean regions, Southern Europe, Central Asia	[41]
5		*L. Donovani*	Post Kala azar dermal leishmaniasis, visceral and cutaneous leishmaniasis	India, Myanmar, Nepal, Sri Lanka, Middle East, China, Ethiopia, Kenya, Sudan	[41]
6		*L. Major*	Cutaneous and mucocutaneous leishmaniasis	Central Asia, Middle East, Central, Northern and West Africa	[42]
7		*L. Mexicana*	Cutaneous and visceral leishmaniasis	United States of America, Venezuela, Ecuador, Peru, Brazil	[40]
8		*L. Venezuelensis*	Cutaneous leishmaniasis	Northern and Southern America, Venezuela	[43]
9		*L. Braziliensis*	Cutaneous and mucocutaneous leishmaniasis	Amazon stretch, Brazil, Southern America, Bolivia, Peru, Venezuela	[44]
10		*L. Guyanensis*	Cutaneous and mucocutaneous leishmaniasis	Southern America, French Guiana, Suriname, Brazil	[45]
11		*L. Panamensis*	Cutaneous and mucocutaneous leishmaniasis	Panama, South and Northern America, Brazil, Ecuador, Columbia, Venezuela	[45]
12		*L. Lainsoni*	Cutaneous leishmaniasis	French Guiana, Peru, Bolivia, Brazil	[46]
13		*L. Naffi*	Cutaneous leishmaniasis	French Guiana, Brazil	[46]
14		*L. Lindenberg*	Cutaneous leishmaniasis	Brazil	[47]
15		*L. Peruviana*	Cutaneous and mucocutaneous leishmaniasis	Peru, Bolivia, Amazon	[48]
16		*L. Shawi*	Cutaneous leishmaniasis	Brazil	[49]
17		*L. Martiniquensis*	Visceral and cutaneous leishmaniasis	Martinique, Thailand, France, Germany, Switzerland, Myanmar	[50]
18		*L. Siamensis*	Visceral and cutaneous leishmaniasis	Central Europe, Thailand, United States of America	[51]
19		*L. Colombiensis*	Visceral and cutaneous leishmaniasis	Columbia	[52]
20	African Trypanosomiasis (aka Sleeping Sickness)	*T. brucei*	Acute and chronic infections	Eastern, Western, Southern and Central Africa	[53]
21	Chagas disease (aka American trypanosomiasis)	*T. cruzi*	Acute and chronic infections	Bolivia, Argentina, Paraguay, Ecuador, El Salvador, Guatemala	[54]

**Table 3:** Drugs/compounds with anti-leishmanial effects identified through a host-directed therapy nanotechnology approach.

Drug/ Compounds	Disease	Target Organism	Model	Outcomes	References
Imatinib	CL	*L. amazonensis*	C57BL/6 mice	A reduction in abrasion in mice and an increase in phagocytosis	[209]
AS101 (tellurium-based compound)	VL	*L. donovani*	BALB/c mice	An increase in the production of ROS and NO. Nuclear factor kappa B (NF-κB) and mitogen-activated protein kinase pathways are activated	[210]
Oleuropein	VL	*L. donovani*	J774A.1 cell line and BALB/c mice	Inflammatory response accompanied by increased levels of interferon-gamma (IFN-γ) and IL-12	[211,212]
Leptin	VL	*L. donovani*	THP-1 cell line	Increase NO production and promote Th1 response to kill parasites	[213]
Mahanine	VL	*L. donovani*	J774A.1 cell line and BALB/c mice	Inhibits the production of Th2 cytokines (IL10) while modulating Th1 cytokines	[214,215]
Simvastatin	CL	*L. major*	BALB/c mice	The drug inhibits cholesterol biosynthesis and reduces promastigotes’ attachment to the host	[206]
Eugenol	CL	*L. amazonensis*	BALB/c mice	The Th1 immune response is triggered by the release of IL-12 and IFN-γ	[216,217]
Phospholipase A2	CL	*L. amazonensis*	BALB/c mice	The stimulation of NF-κB in macrophages and tumor necrosis factor-alpha (TNF-alpha) production (increased Th1 immune response) and NO production is also enhanced	[218]

Reproduced with permission from [203].

**Table 4:** Drugs/compounds with anti-leishmanial effects that were identified through drug repurposing.

Drug/Compounds	Disease	Target Organism	Model	Outcomes	References
Sertraline	VL	*L. infantum*	BALB/c mice	Reduced the parasite’s growth significantly. In addition to oxidative damage, essential metabolic pathways were altered	[226,227]
Cladribine, Lamivudine, Metformin, Perphenazine, Rifabutin, and Tenofovir	CL	*L. braziliensis* and *L.* *panamensis*	U-937 cell line	According to in vitro studies, rifabutin and perphenazine inhibited parasites better than the other drugs	[228]
Gold (I) triphenyl- phosphine and triethyl- phosphine based complexes	CL	*L. amazonensis* and *L. braziliensis*	BALB/c mice	It is reported that the IC_50_ for the anti-leishmanial activity is 0.5–5.5 μM. ROS-mediated cell death is caused by the inhibition of trypanothione reductase activity	[229]
Triclosan	CL	*L. amazonensis*	BALB/c mice	The parasite was inhibited in its growth, as well as mitochondria were damaged and membrane permeability was reduced	[230]
Simeprevir	VL	*L. donovani*	THP-1 cell line	ROS-mediated cell death combined with leishmanicidal properties against Promastigotes and Amastigotes	[231]
Rapamycin	CL	*L. major* and *L. tropica*	BALB/c mice	Proliferation of the parasite was markedly inhibited. A reduction in parasite load in mice along with the polarization of the immune system towards Th1 was observed	[232]
Nifuratel	CL and VL	*L. major*, *L. infantum*, and *L. donovani*	BALB/c mice	In a mouse model, both oral and intralesional administration cured cutaneous and visceral infections	[233]

Reproduced with permission from [203].

**Table 5:** Drugs/compounds with anti-leishmanial effects identified through nanotechnology approach.

Drug/Compounds	Disease	Target Organism	Model	Outcomes	References
Silver NPs containing Fig and Olive extracts	CL	*L. major*	BALB/c mice	Reduction in skin lesions and improved antioxidative capacity	[248]
Nano-hydrogels loaded with buparvaquone	CL	*L. amazonensis*	BALB/c mice	Infected BALB/c mice showed a significant decrease in parasitic burden of 95%	[249]
Chitosan/CdO core shell nanodots	CL	*L. major*	THP-1 cell line	The compound inhibited promastigotes proliferation and induced Th1 immunity at IC_50_ 0.6 μL/mL	[250]
Cyclodextrin NPs containing Amp B and paromomycin	VL	*L. donovani*	J774A.1 cell line and Swiss albino mice	By inhibiting parasite growth with an IC_50_ 0.013 μg/mL, it reduced the parasitic burden by 70–90%	[251]
Chitosan NPs containing Amp B and paromomycin	VL	*L. donovani*	J774A.1 cell line	NPs inhibit parasite growth better than Amp B alone, and >90% parasite clearance was reported	[252]
Nanotube appended with Amp B	VL	*L. donovani*	J774A.1 cell line and golden hamster	Composite graphene–carbon nanotubes significantly reduced parasitic proliferation by >90% when compared to AmB alone, proving that it is a safe cure for parasites	[253]
Guar gum NPs containing and Amp B	VL	*L. donovani*	Golden hamster	It inhibits parasites by 2–3 fold compared to drug alone and reduces parasitic burden by 95%	[254]

Reproduced with permission from [203].

**Table 6:** Lists of novel anti-leishmanial drugs that target specific biochemical pathways.

Drug Candidate	Drug Target	Mode of Action	References
Artesunate Quinine Mefloquine	GAPDH	Inhibits the parasites’ glycolytic enzymes GAPDH	[261,262]
Cycloguanil	DHFR	Inhibits DHFR	[263,264,265]
Trimethoprim (TMP, 2)			
ZINC57774418 (Z18)			
ZINC69844431 (Z31)			
ZINC71746025 (Z25)			
D11596 (DB96)		Inhibits DHFR activity	[265]
2-(4-((2,4- dichlorobenzyl)oxy)phenyl)-1Hbenzo[d]imidazole	DHFR and PTR1	DHFR-TS/PTR1 inhibitors	[266]
2-(4-((2,4- dichlorobenzyl)oxy)phenyl)-1Hbenzo[d]imidazole-1Hbenzo[d]oxazole	DHFR and PTR1		
Trichloro[1,2-ethanediolato-O,O’]tellurate (AS101)	TR	Induces ROS-mediated apoptosis by binding to TR cysteine residues	[210]
β-sitosterol CCL		Inhibit TR activity	[267]
Hypericin	Spermidine synthase	ROS and spermidine reduction	[268,269]

Reproduced with permission from [4].

**Table 7:** Studies involving advanced peptides against leishmaniasis and Chagas disease.

Peptide	Source	Sequence	IC_50_ µg/mL	Study Model	Reference
NK-2	Synthetic peptide	KILRGVCKKIMRTFLRRISKDILTGKK	-	in vitro	[281]
Temporizin-1	Synthetic peptide	FLPLWLWLWLWLWKLK	-	in vitro	[282]
Defensin-α1	*Homo sapiens* (Human)	ACYCRIPACIAGERRYGTCIYQGRLWAFCC	-	in vitro	[283]
Phylloseptin 7	*Phyllomedusa nordestina* (Frog)	FLSLIPHAINAVSAIAKHF	0.34	in vitro	[284]
DS 01	*Phyllomedusa oreades* (Frog)	GLWSTIKQKGKEAAIAAAKAAGQAALGAL	-	in vitro	[285]
Melittin	*Apis mellifera* (Bee)	-	2.44	in vitro	[286]
Polybia-CP	*Polybia paulista* (Wasp)	ILGTILGLLSKL	-	in vitro	[287]
Hmc 364–382	*Penaeus monodon* (Shrimp)	NVQYYGALHNTAHIVLGRQ	4.79	in vitro	[287]

Reproduced with permission from [288].

**Table 8:** Structural analysis of selected peptide profiles for leishmaniasis and Chagas disease.

Species (Diseases)	Peptide Name	Sources	Sequences	Study Model	Net Charge	Hydrophobicity (kcal/mol)	PI	Length	References
Leishmania	DRS 01	Phyllomedusa	GLWSTIKQKGKEAAIAAAKAAGQAALGAL	in vitro	+3	+26.50	10.60	29	[318]
Leishmania	Dermaseptin 4	Phyllomedusa	GLWSTIKQKGKEAAIAAAKAAGKAALNAASEAL	in vitro	+3	+33.32	10.43	33	[284]
Leishmania	Dermaseptin 1	Nordestina	GLWSTIKNVGKEAAIAAGKAALGAL	in vitro	+2	+21.05	10.37	25	[284]
Leishmania	p-Acl	Agkistrodon contortrix	KKYKAYFKFKCKK	in vitro	+7	+23.14	10.45	13	[319]
Leishmania	p-AclR7	Laticinctus	RRYRAYFRFRCRR	in vitro	+7	+16.21	12.36	13	[319]
Leishmania	Eumenitin-R	Eumenes rubrofemoratus	LNLKGLIKKVASLLN	in vitro	+3	+12.28	10.89	15	[320]
Leishmania	Dermaseptin-01	Amphibian	GLWSTIKNVGKEAAIAAGKAALGAL	*-*	+2	+21.05	10.35	25	[321]
Leishmania	Dermaseptin-H3	Amphibian	GLWSTIKNVGEAAIAAGKAALGAL	*-*	+1	+18.25	9.95	24	[321]
Leishmania	Melittin	Insect	GIGAVLKVLTTGLPALISWIKRKQQ	*-*	+4	+13.83	11.79	24	[322]
Leishmania	Melittin	Insect	GIGAVLTTGLPALISWIKRKRQQ	*-*	+4	+14.55	12.51	23	[322,323]
Leishmania	Phylloseptin-1	Amphibian	FLSLIPHAINAVSAIAKHN	*-*	+1	+12.09	9.93	19	[324]
Leishmania	Bombinin H	Amphibian	IIGPVLGLVGSALGGLLKKI	*-*	+2	+9.82	10.65	20	[325]
Leishmania	LL-37	Cathelicidin	LLGDFFRKSKEKIGKEFKRIVQRIKDFLRNLVPRTES	in vitro	+6	+41.03	11.15	37	[326]
Leishmania	E6	Synthetic	RRWRIVVIRVRR	in vitro	+6	+13.05	13.18	12	[326]
Leishmania	cecropin-A	Hemolymph of the giant silkworm Hyalophora cecropia	KWKLFKKIEKVGQNIRDGIIKAGPAVAWVGQATQIAK	in vitro	+6	+33.11	10.94	37	[327]
Leishmania	Cecropin-D	Galleria mellonella hemolymph	ENFFKEIERAGQRIRDAIISAAPAVETLAQAQKIIKGGD	in vitro	0	+42.64	7.07	39	[323]
Leishmania	Eumenitin-F		LNLKGIFKKVASLLT	in vitro	+3	+11.22	10.89	15	[303]
Leishmania	Eumenitin-R		LNLKGLIKKVASLLN	in vitro	+3	+12.28	10.89	15	[303]
Leishmania	P1	Direct screening of a linear hexa-peptide library on *L. major* metacyclic parasite	MASKPQR	in vitro and in vivo	+2	+13.71	11.53	7	[328]
Leishmania	P2	Direct screening of a linear hexa-peptide library on *L. major* metacyclic parasite	MAAKYN	in vitro and in vivo	+1	+11.17	9.58	6	[328]
Leishmania	P3	Direct screening of a linear hexa-peptide library on *L. major* metacyclic parasite	MAHYSG	in vitro and in vivo	0	+10.96	7.63	6	[328]
Leishmania	P4	Direct screening of a linear hexa-peptide library on *L. major* metacyclic parasite	MYVIRG	in vitro and in vivo	+1	+7.90	9.68	6	[328]
Leishmania	P5	Direct screening of a linear hexa-peptide library on *L. major* metacyclic parasite	SLSWVC	in vitro and in vivo	0	+5.00	4.93	6	[328]
Leishmania	P6	Direct screening of a linear hexa-peptide library on *L. major* metacyclic parasite	QRKMAS	in vitro and in vivo	+2	+13.57	11.52	6	[328]
Chagas disease		Mucin-associated surface proteins (MASP)	SLLSDAENPGGEVFNDNK	in vitro	−3	+26.98	3.53	18	[328]
Chagas disease		Mucin-associated surface proteins (MASP)	DAENPGGEVFNDNKKGLSRV	in vitro	−1	+33.07	4.48	20	[328]

## Data Availability

Data can be found within the article.
